# Efficacy and safety of concomitant immunotherapy and denosumab in patients with advanced non-small cell lung cancer carrying bone metastases: A retrospective chart review

**DOI:** 10.3389/fimmu.2022.908436

**Published:** 2022-08-29

**Authors:** Hong-Shuai Li, Si-Yu Lei, Jun-Ling Li, Pu-Yuan Xing, Xue-Zhi Hao, Fei Xu, Hai-Yan Xu, Yan Wang

**Affiliations:** ^1^ Department of Medical Oncology, National Cancer Center/National Clinical Research Center for Cancer/Cancer Hospital, Chinese Academy of Medical Sciences and Peking Union Medical College, Beijing, China; ^2^ Department of Comprehensive Oncology, National Cancer Center/National Clinical Research Center for Cancer/Cancer Hospital, Chinese Academy of Medical Sciences and Peking Union Medical College, Beijing, China

**Keywords:** immunotherapy, non-small cell lung cancer, bone metastases, efficacy, safety, denosumab, synergistic efficacy

## Abstract

**Background:**

Synergistic anti-tumor effects were observed *in vivo* and *in vitro* when immune checkpoint inhibitors (ICIs) were combined with denosumab. However, the clinical benefit and safety of this synergy have not been adequately evaluated in non-small cell lung cancer (NSCLC).

**Methods:**

Consecutive charts of NSCLC patients with bone metastases between December 2020 and December 2021 in the Chinese National Cancer Center were reviewed. The entire cohort was divided into one experimental group (denosumab + ICIs [DI]) and three control groups (denosumab + non-ICIs [DnI], phosphates + ICIs [PI], phosphates + non-ICIs [PnI]). Real-world objective response rates (ORRs), median progression-free survival (mPFS), skeletal-related events (SREs), and adverse events (AEs) were compared between groups.

**Results:**

A total of 171/410 (41.7%) patients with advanced or recurrent NSCLC carrying bone metastases who received bone-targeted therapy were eligible for analysis. Although the DI group showed a better benefit trend, differences were not statistically significant concerning the therapeutic efficacy among the DI group (n = 40), PI group (n = 74), DnI group (n = 15), and PnI group (n = 42) (ORRs: 47.5%, 43.2%, 33.3%, and 40.5%, respectively, *p* = 0.799; and mPFS: 378, 190, 170, and 172 days, respectively, *p* = 0.115; SREs: 5%, 10.8%, 13.3%, and 11.9%, respectively, *p* = 0.733). Nevertheless, further analysis in the NON-DRIVER cohort revealed a greater benefit for the DI group (*p* = 0.045). Additionally, the AEs of the DI group were not significantly different from those of the PI, DnI, and PnI groups (AEs: 27.5%, 39.2%, 26.7%, and 28.6%, respectively, *p* = 0.742). Furthermore, the multivariate analysis revealed the independent prognostic role of DI treatment for PFS in the overall cohort. Within the DI group, we did not observe differences in benefit among different mutational subgroups (*p* = 0.814), but patients with single-site bone metastasis (*p* = 0.319) and high PD-L1 expression (*p* = 0.100) appeared to benefit more, though no significant differences were observed.

**Conclusions:**

Denosumab exhibited synergistic antitumor efficacy without increasing toxicity when used concomitantly with ICIs in patients with advanced non-small cell lung cancer carrying bone metastases.

## 1. Introduction

In recent years, lung cancer incidence and mortality rates have remained high as the aging population has intensified, along with the effects of industrialization and air pollution (1). As the main body of lung cancer, the 5-year overall survival (OS) rate of metastatic non-small cell lung cancer (NSCLC) patients is only 5% (2). In the past 20 years, the treatment of lung cancer has undergone radical changes, especially with the in-depth development of the molecular pathology of lung cancer and the rise of immunotherapy, including monoclonal antibodies (mAbs) blocking programmed cell death 1 (PD-1)/PD1 ligand 1 (PD-L1) and the cytotoxic T-lymphocyte-associated antigen 4 (CTLA-4), known as immune checkpoint inhibitors (ICIs). In the renowned KEYNOTE-024 trial, pembrolizumab obtained a 5-year OS rate of 31.9%, which is granted as an effective first-line treatment option for NSCLC patients with PD-L1 TPS ≥50% by the FDA (3). However, ICI resistance is a challenge that we must embrace. To overcome the resistance and expand the population benefiting from ICIs, non-redundant mechanisms of tumor-induced immunosuppression need to be explored, and combinatory therapy is expected to be more effective (4).

Receptor activator of nuclear factor­κB ligand (RANKL, also called TNFSF11) is a member of the tumor necrosis factor (TNF) superfamily and a ligand both for RANK (also called TNFRSF11A) and osteoprotegerin (OPG, also called TNFRSF11B) (5). The RANK–RANKL–OPG axis is essential for physiological bone resorption and destruction, and it also plays an important role in pathological states such as osteoporosis and bone destruction at the foci of bone metastases (2, 5–7). As the first fully human anti-RANKL mAb, denosumab was demonstrated to be non-inferior to zoledronic acid (ZA) in delaying time to the first on-study skeletal-related events (SREs) in a randomized, double-blind study enrolling multiple advanced cancer types (including lung cancer) (8) and has been approved by the FDA for preventing SREs in solid tumors. Unexpectedly, the exploratory analysis also revealed an OS benefit of denosumab over ZA in NSCLC patients with bone metastases (hazard ratio [HR] = 0.78, 9.5 vs. 8.0 months; *p* = 0.01) (9).

Increasing evidence indicates that the survival benefit may stem from the synergistic anti-tumor effects of the combination of ICIs and denosumab (10–14). Series studies conducted by Ahern et al. revealed *via* a mouse model, that the combination of RANKL inhibitor and ICIs significantly increased the number of infiltrating T cells and expression of anti-tumor cytokines (IFN-γ, etc.) in the tumor microenvironment (TME) compared to a single agent, and the combination therapy significantly reduced mouse tumor burden (11, 12).

Recently, several retrospective studies have suggested the feasibility of this combination regimen in advanced NSCLC patients with bone metastases (14–18). However, the findings of these studies need further confirmation due to the lack of suitable control groups and the presence of confounding factors. This study evaluated the efficacy and safety of the combination of ICIs and denosumab for advanced NSCLC patients with bone metastases in a real-world setting.

## 2. Methods

### 2.1. Study design and rationale

**Figure 1:**
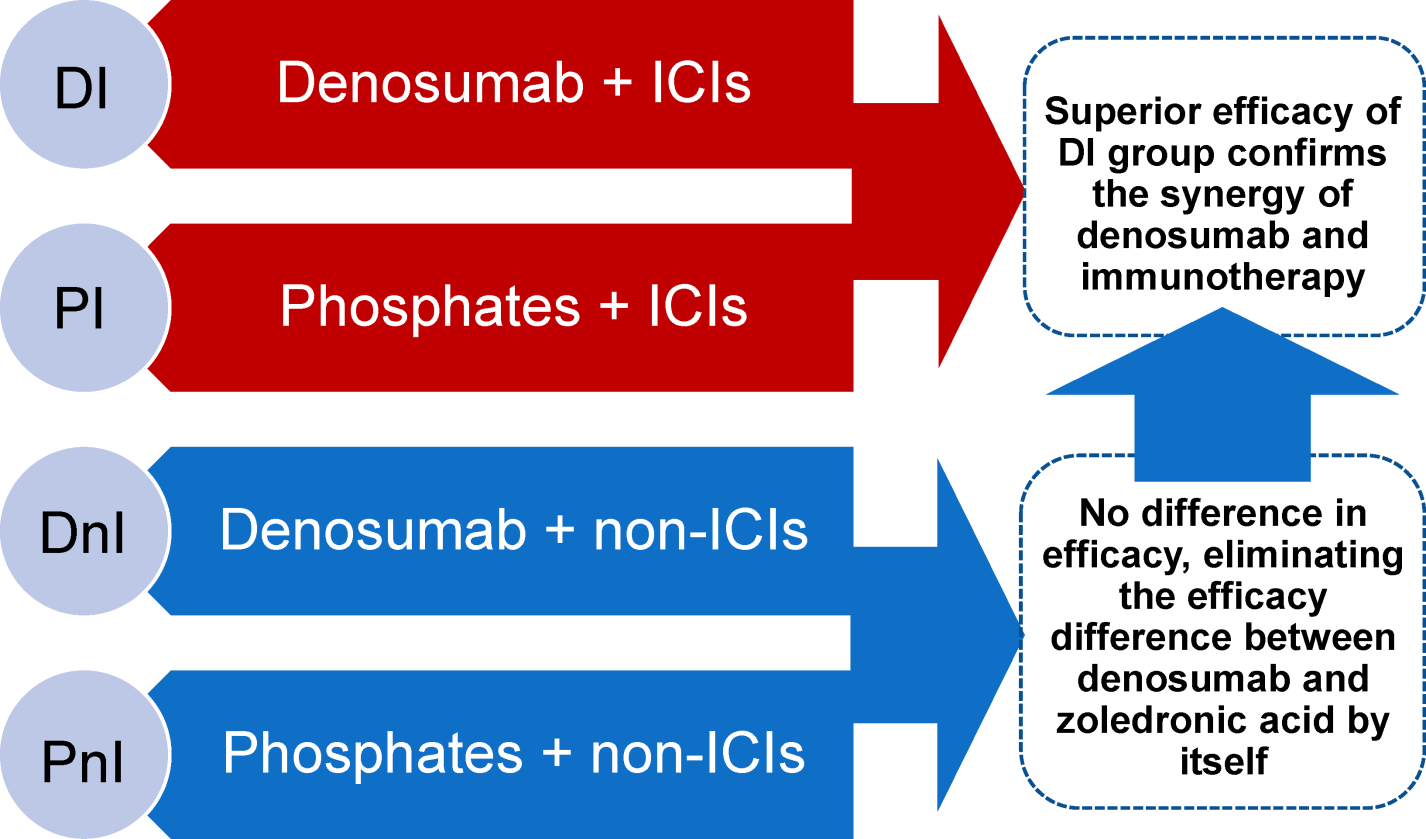
Study design and rationale.

A retrospective, observational chart review was conducted on NSCLC patients with bone metastases who were enrolled in the Chinese National Cancer Center between December 2020 and December 2021. To fully assess the synergistic effects of denosumab and ICIs, based on the therapeutic pattern of systematic therapy and bone-targeted therapy (BTT), the entire cohort was divided into one experimental group (denosumab + ICIs [DI]) and three control groups (denosumab + non-ICIs [DnI], phosphates + ICIs [PI], phosphates + non-ICIs [PnI]). Real-world objective response rates (ORRs), median progression-free survival (mPFS), adverse events (AEs), and SREs were planned to be compared between groups. The DnI and PnI groups were set up to verify whether a difference in efficacy existed between denosumab and phosphates in the absence of ICIs (in the context of no synergistic condition existing), thus establishing a baseline for comparison between the DI and PI groups. On this basis, a synergistic effect of DI treatment would be confirmed if the efficacy of the DI group was better than that of the PI group (**Figure 1**).

### 2.2. Patient eligibility, grouping, and data collection

Patients diagnosed with NSCLC who have received chemotherapy either alone or along with ICI (pembrolizumab, nivolumab, atezolizumab, sintilimab, or camrelizumab) as well as concomitant BTT (phosphates [including zoledronic acid, pamidronate disodium, or ibandronate monosodium] or denosumab) were identified. Concomitant therapy was defined as receipt of BTT at any point before systematic therapy (chemotherapy combined with ICI or not) initiation, or no later than 30 days following systematic therapy initiated at least 4 months before the data cutoff (31 December 2021). Demographics, clinicopathological information, molecular features, and detailed treatment history data were extracted from electronic medical records. Patients with too much key clinical information missing were excluded.

Sub-cohorts were defined during the data analysis. The NON-DRIVER cohort included cases without *EGFR*, *HER-2*, *ALK*, *ROS1*, *MET*, *RET*, and *BRAF* mutations, except for *KRAS* mutations. The WILD-TYPE cohort included cases without *EGFR*, *HER-2*, *ALK*, *ROS1*, *MET*, *RET*, *BRAF*, or *KRAS* mutations.

All charts were reviewed by the primary author, the confidentiality of all patients was maintained by assigning each patient a study number, and all data were securely stored in the hospital. The study was conducted in accordance with the Declaration of Helsinki (as revised in 2013). Institutional Review Board approval of the study protocol was obtained (No.: NCC-008296) before study conduct and informed patient consent was waived as this was a retrospective study.

### 2.3. Treatment and efficacy/toxicity evaluation

In this real-world study, denosumab was administered subcutaneously at 120 mg approximately every 28 days, while phosphates were administered intravenously approximately every 21 days. The PD-1 or PD-L1 inhibitor was administered by intravenous injection approximately every 3 weeks, and the specific dosage was determined according to the specific drug instructions. Phosphates were generally administered within three days of the administration of ICIs. Patient compliance was confirmed from the electronic medical records.

Real-world tumor response was analyzed and produced by trained extractors following a pre-defined process, including an integrated assessment of radiologist reports and clinical documentation data. The frequency of imaging review to assess response was every 6–8 weeks in a real-world setting. The objective tumor response was determined according to the Response Evaluation Criteria in Solid Tumors (RECIST 1.1) guidelines (19). The objective response was divided into two categories: the objective response was divided into complete response (CR) and partial response (PR), while the disease control was divided into CR, PR, or stable disease (SD).

Toxicity was assessed according to the Common Terminology Criteria for Adverse Events (version 5.0). Acute phase AEs such as flu-like reactions, including fever, myalgia, and chills, were counted only as treatment-related if they occurred within 24 h of phosphate infusion; otherwise, they were not counted as AEs to BTT.

### 2.4. Statistical analysis

Categorical variables were reported as absolute numbers and percentages. The chi-square test was used for comparisons between different groups. The data cut-off date was 28 February 2022, when the disease status of the patients was confirmed. PFS was defined as the time from concomitant administration to disease progression or death from any cause. Patients who were lost to follow-up were judged to be censored and the last determinable time of survival was used as the time of termination of follow-up. The relationship between various variables and survival was evaluated using the Kaplan–Meier method. Differences between survival curves were tested for statistical significance using the Log-rank test. Significant prognostic predictors for patients identified by univariate analyses were further assessed by multivariate analyses using the Cox proportional hazards regression model. Statistical analyses were performed, and analytic graphs were created using GraphPad Prism 8 software (GraphPad Software, San Diego, CA, USA). An α value of 0.05 was used as the examination standard.

## 3. Results

### 3.1 Baseline characteristics

**Figure 2:**
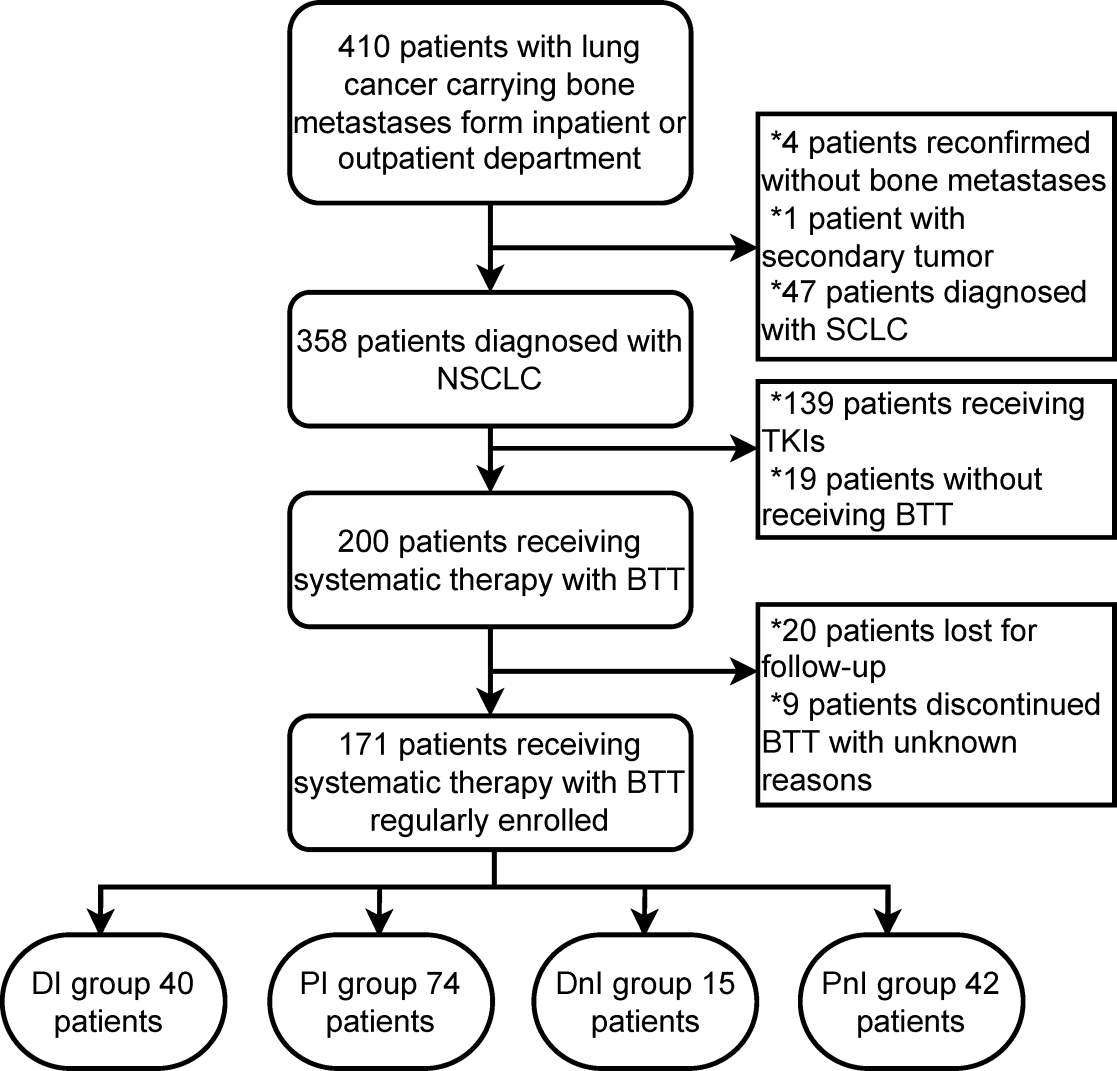
Flow chart of patient selection. NSCLC, non-small cell lung cancer; SCLC, small-cell lung cancer; BTT, bone-targeted therapy; TKIs, tyrosine-kinase inhibitors; DI, denosumab + ICIs; DnI, denosumab + non-ICIs; PI, phosphates + ICIs; PnI, phosphates + non-ICIs.

In total, 171/410 (41.7%) patients with NSCLC carrying bone metastases who were treated with BTT were enrolled at the Chinese National Cancer Center between December 2020 and December 2021 **(** **Figure 2** **)**. Based on different treatment combinations of systematic therapy and BTT, the overall cohort was divided into 4 groups: DI (n = 40), PI (n = 74), DnI (n = 15), and PnI (n = 42). The baseline characteristics of the four groups are displayed in **Table 1**.** A** higher proportion of adenocarcinoma histology was observed in the DnI and PnI groups (*p* = 0.038), while a higher proportion of PD-L1 expression in the DI and PI groups (*p* = 0.010) was observed, and the highest proportion of *KRAS* mutation in the DI group (*p* = 0.145) was revealed, despite a significant difference being unreached. No statistically significant differences were observed for other baseline characteristics.

**Table 1:** Baseline characteristics between four treatment subgroups (n = 171).

Characteristics	Treatment modality/n (%)				p^&^
	DI group (n = 40)	PI group (n = 74)	DnI group (n = 15)	PnI group (n = 42)
**Age**					0.444
<60	21 (52.5)	33 (44.6)	10 (66.7)	20 (47.6)	
≥60	19 (47.5)	41 (55.4)	5 (33.3)	22 (52.4)	
**Gender**					0.217
Female	5 (12.5)	15 (20.3)	5 (33.3)	12 (28.6)	
Male	35 (87.5)	59 (79.7)	10 (66.7)	30 (71.4)	
**Smoking history^#^**					0.614
Never smoker	10 (25)	18 (24.3)	7 (46.7)	14 (33.3)	
Ever smoker	5 (12.5)	9 (12.2)	1 (6.7)	3 (7.1)	
Current smoker	24 (60)	44 (59.5)	6 (40)	21 (50)	
Unknown	1 (2.5)	3 (4.1)	1 (6.7)	4 (9.5)	
**DM/HT history**					0.485
No	26 (65)	44 (59.5)	10 (66.7)	31 (73.8)	
Yes	14 (35)	30 (40.5)	5 (33.3)	11 (26.2)	
**Histology**					**0.038**
AC	31 (77.5)	51 (68.9)	14 (93.3)	37 (88.1)	
SCC	5 (12.5)	18 (24.3)	1 (6.7)	1 (2.4)	
Others*	4 (10)	5 (6.8)	0	4 (9.5)	
**Grade**					0.211
Well differentiated	2 (5)	0	0	0	
Moderately differentiated	2 (5)	11 (14.9)	1 (6.7)	3 (7.1)	
Poorly differentiated	15 (37.5)	34 (45.9)	4 (26.7)	19 (45.2)	
Undifferentiated	2 (5)	1 (1.4)	0	1 (2.4)	
Unknown	19 (47.5)	28 (37.8)	10 (66.7)	19 (45.2)	
**Mutation status**					0.145
*ALK*	0	0	0	1 (2.4)	
*ROS1*	0	0	0	1 (2.4)	
*MET*	0	1 (1.4)	0	0	
*RET*	1 (2.5)	0	0	1 (2.4)	
*BRAF*	2 (5)	3 (4.1)	0	3 (7.1)	
*HER2*	2 (5)	2 (2.7)	2 (13.3)	3 (7.1)	
*EGFR*	7 (17.5)	8 (10.8)	5 (33.3)	10 (23.8)	
*KRAS*	16 (40)	21 (28.4)	4 (26.7)	11 (26.2)	
Wild-type	12 (30)	39 (52.7)	4 (26.7)	12 (28.6)	
***TP53* co-mutation**					0.200
No	24 (60)	57 (77)	12 (80)	32 (76.2)	
Yes	16 (40)	17 (23)	3 (20)	10 (23.8)	
**PD-L1 level**					**0.010**
<1%	6 (15)	20 (27)	9 (70)	12 (28.6)	
1%-49%	10 (25)	10 (13.5)	1 (6.7)	10 (23.8)	
≥50%	12 (30)	16 (21.6)	0	3 (7.1)	
Unknown	12 (30)	28 (37.8)	5 (33.3)	17 (40.5)	
**Brain metastases**					0.338
No	30 (75)	64 (86.5)	12 (80)	37 (88.1)	
Yes	10 (25)	10 (13.5)	3 (20)	5 (11.9)	
**Bone metastases**					0.457
Single	6 (15)	19 (25.7)	4 (26.7)	7 (16.7)	
Multiple	34 (85)	55 (74.3)	11 (73.3)	35 (83.3)	
**Visceral metastases**					0.543
No	27 (67.5)	49 (66.2)	10 (66.7)	33 (78.6)	
Yes	13 (32.5)	25 (33.8)	5 (33.3)	9 (21.4)	
**Application line**					0.255
1	27 (67.5)	57 (77)	11 (73.3)	36 (85.7)	
2	8 (20)	14 (18.9)	3 (20)	4 (9.5)	
3	4 (10)	3 (4.1)	0	2 (4.8)	
4	1 (2.5)	0	1 (6.7)	0	
**ECOG PS**					0.294
0	11 (27.5)	10 (13.5)	4 (26.7)	9 (21.4)	
1	27 (67.5)	55 (74.3)	10 (66.7)	32 (76.2)	
2	2 (5)	9 (12.2)	1 (6.7)	1 (2.4)	

DI, denosumab + ICIs; DnI, denosumab + non-ICIs; PI, phosphates + ICIs; PnI, phosphates + non-ICIs; DM, diabetes mellitus; HT, hypertension; AC, adenocarcinoma; SCC, squamous cell carcinoma; ALK, anaplastic lymphoma receptor tyrosine kinase; ROS1, ROS proto-oncogene 1, receptor tyrosine kinase; MET, MET proto-oncogene, receptor tyrosine kinase; RET, ret proto-oncogene; BRAF, B-Raf proto-oncogene, serine/threonine kinase; HER2, erb-b2 receptor tyrosine kinase 2; EGFR, epidermal growth factor receptor; KRAS, Kirsten Rat Sarcoma Viral Oncogene Homolog; PD-L1, programmed death-ligand 1; ECOG PS, Eastern Cooperative Oncology Group performance status. *Current smoker refers to someone who has smoked more than 100 cigarettes (including hand-rolled cigarettes, cigars, cigarillos, etc.) in their lifetime and has smoked in the last 28 days. Ever smoker refers to someone who has smoked more than 100 cigarettes in their lifetime but has not smoked in the last 28 days. Never smoker is someone who has not smoked more than 100 cigarettes in their lifetime and does not currently smoke. ^&^The chi-square test was employed for the comparative analysis. ^#^Including large cell neuroendocrine carcinoma, sarcomatoid carcinoma, and adenosquamous carcinoma.Bold values indicate that the differences are statistically significant.

For the DI group, a predominant proportion of males (87.5%) was observed. More than half (60%) of patients were current smokers, and the majority of patients (77.5%) had adenocarcinoma histology. Non-driver patients (including *KRAS*-mutated and wild-type ones) account for most of the DI group. Nearly 70% of patients initiated BTT along with the first-line systematic therapy. Most patients had multiple bone metastases (85%) and a PS status of 1 (67.5%) **(** **Table 1** **)**.

### 3.2 Efficacy evaluation

**Figure 3:**
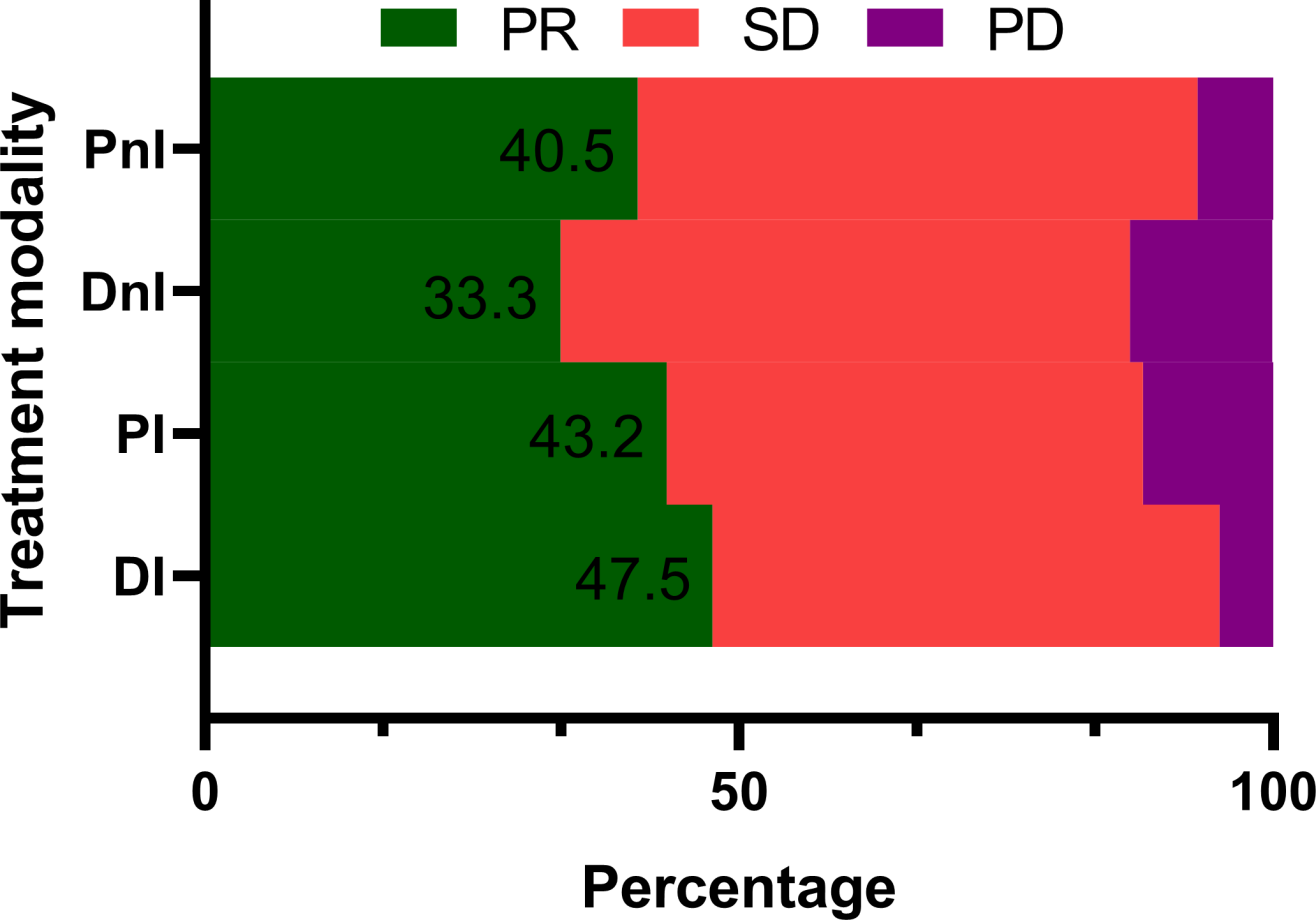
Treatment responses of different treatment modalities (n = 171). PR, partial response; SD, stable disease; PD, progressive disease; DI, denosumab + ICIs; DnI, denosumab + non-ICIs; PI, phosphates + ICIs; PnI, phosphates + non-ICIs.

**Figure 4:**
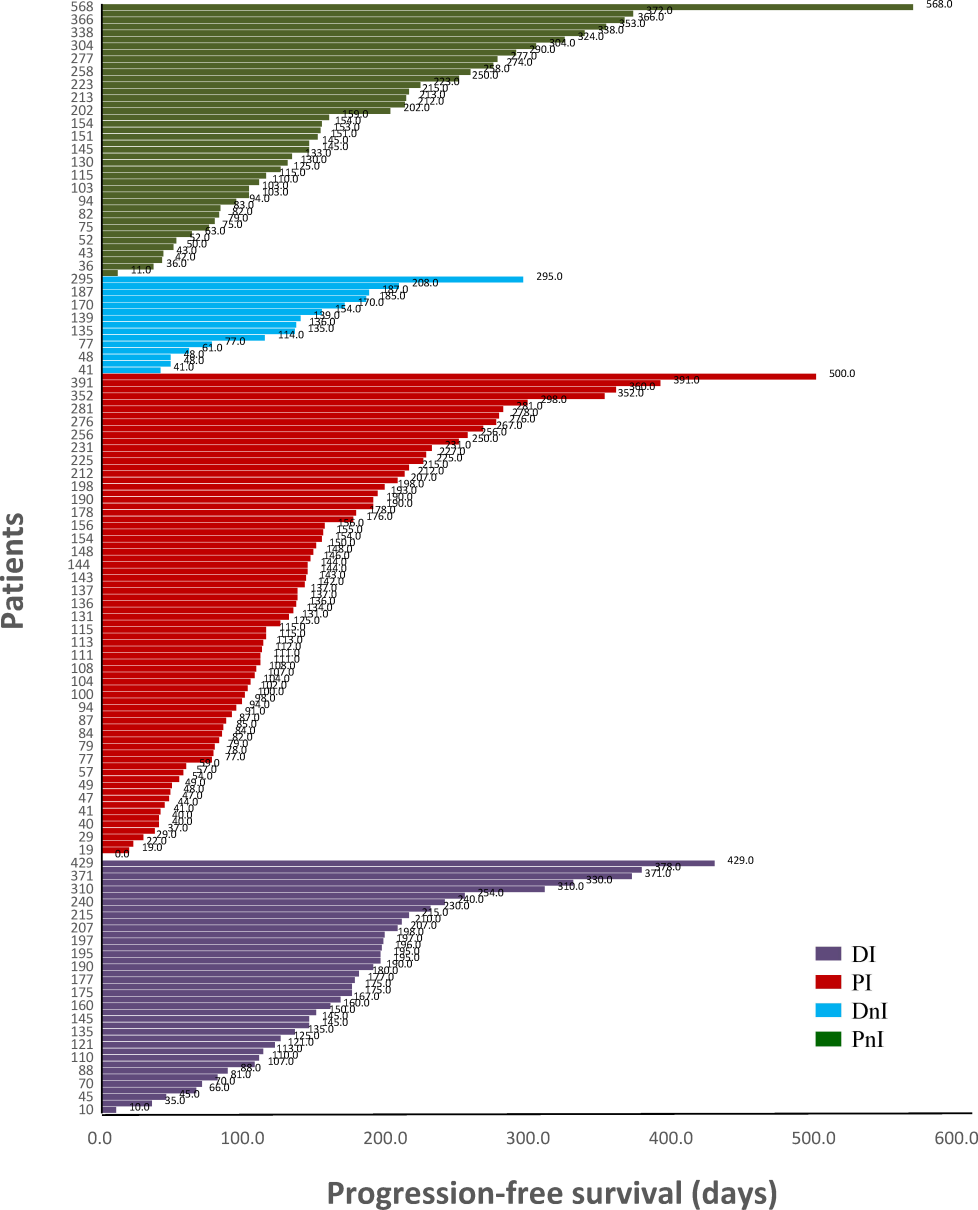
Swimming plot of different treatment modalities (n = 171). DI, denosumab + ICIs; DnI, denosumab + non-ICIs; PI, phosphates + ICIs; PnI, phosphates + non-ICIs.

Among the 40 evaluable patients in the DI group, 19 (47.5%), 19 (47.5%), and two (5%) had PR, SD, and *de novo* resistance to DI treatment, respectively. The ORR was 47.5% and the disease control rate (DCR) was 95% (**Figure 3**). At the data cut-off date, the mPFS was 378 days (95% CI, 118.5–636.5 days), and the median follow-up duration was 198 days (95% CI, 181.6–214.4 days) in the DI group. The PFS was mature in 14 (35%) patients, and the tumors of 26 patients were still under control (**Figure 4**).

**Figure 5:**
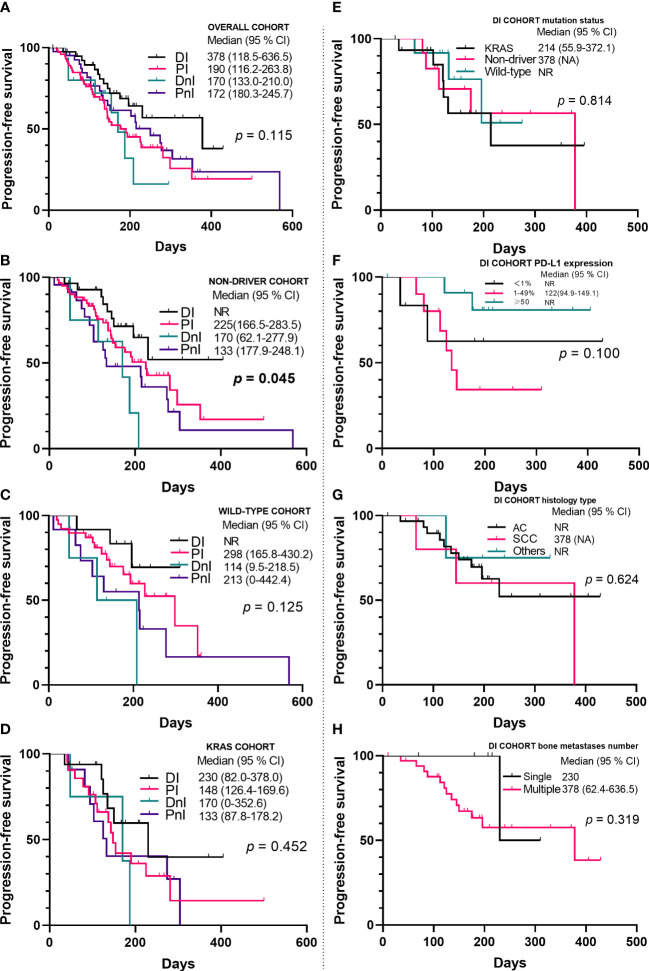
Kaplan–Meier analyses of progression-free survival in the overall cohort **(A–D)** (n = 171) and the DI cohort **(E–H)** (n = 40). NR, not reached; PD-L1, programmed death ligand-1; AC, adenocarcinoma; SCC, squamous cell carcinoma; DI, denosumab + ICIs; DnI, denosumab + non-ICIs; PI, phosphates + ICIs; PnI, phosphates + non-ICIs.

By contrast, the DI group showed a trend for better ORR (**Figure 3**) and mPFS (**Figure 5A**) than those of the PI, DnI, and PnI groups (ORRs: 47.5%, 43.2%, 33.3%, and 40.5%, respectively, *p* = 0.799; and mPFS: 378, 190, 170, and 172 days, respectively, *p* = 0.115), though the differences were not statistically significant. To exclude the confounding effect of driver genes on efficacy, we extracted the NON-DRIVER (including *KRAS*-mutated and wild-type cases) cohort, WILD-TYPE cohort, and *KRAS* cohort from the overall cohort. Kaplan–Meier analysis in the NON-DRIVER cohort confirmed a statistically significant benefit for the DI group over the control groups (mPFS: NR, 225, 170, and 133 days, respectively, *p* = 0.045) (**Figure 5B**). In the WILD-TYPE cohort, a more pronounced benefit for the DI group appeared to be observed, but due to the reduced cohort scale, there was insufficient statistical power to demonstrate a statistically significant difference (*p* = 0.125) (**Figure 5C**). In the *KRAS* cohort, the DI group also showed a trend for better therapeutic efficacy than that of the control groups (mPFS: 230, 148, 170, and 133 days, respectively). Nevertheless, the advantage of the mPFS of the DI group was less obvious (*p* = 0.452) (**Figure 5D**).

With regards to SRE prevention, the DI group demonstrated a trend for a lower SRE rate than that of the PI, DnI, and PnI groups (5%, 10.8%, 13.3%, and 11.9%, respectively), though a significant difference was not reached (*p* = 0.733) **(** **Table 2** **)**.

**Table 2:** Skeletal-related events between four treatment subgroups (n = 171).

SREs	Treatment modality/n (%)				Total/n (%)*
	DI group (n = 40)	PI group (n = 74)	DnI group (n = 15)	PnI group (n = 42)
Pathologic fractures	1 (2.5)	1 (1.4)	0	1 (2.4)	3 (1.8)
Bone surgery	0	1 (1.4)	0	0	1 (0.6)
Bone radiotherapy	1 (2.5)	5 (6.8)	2 (13.3)	4 (9.5)	12 (7.0)
Spinal cord compression	0	1 (1.4)	0	0	1 (0.6)
Malignant hypercalcemia	0	0	0	0	0
**Total/n (%)**	2 (5)	8 (10.8)	2 (13.3)	5 (11.9)	17 (9.9)

DI, denosumab + ICIs; DnI, denosumab + non-ICIs; PI, phosphates + ICIs; PnI, phosphates + non-ICIs; SREs, skeletal-related events. *Calculated as the percentage of the overall cohort.

### 3.3 Survival analysis

To determine the influence of different variates on prognosis, we conducted univariate and multivariate analyses (**Table 3**) for the whole cohort. In the univariate analysis, visceral metastases (*p* = 0.021), application line (*p* = 0.006), and Eastern Cooperative Oncology Group performance status (ECOG PS) (*p* = 0.001) were all statistically significant prognostic factors for PFS. In the multivariate analysis, mutation status (*p* = 0.043), PD-L1 expression level (*p* = 0.036), application line (*p* = 0.011), ECOG PS (*p* = 0.021), and treatment modality (*p* = 0.042) were independent predictors of PFS (**Table 3**). Specifically, we also examined the effect of different variables on the efficacy within the DI group (**Figures 5E–H**). We did not observe differences in benefit among different mutational subgroups (*p* = 0.814), but patients with single-site bone metastasis (*p* = 0.319) and high PD-L1 expression (*p* = 0.100) appeared to benefit more, though no significant differences were observed.

**Table 3:** Univariate and multivariate analyses of PFS in the whole cohort (n = 171).

Variables	n	Univariate analysis*		Multivariate analysis^#^		
		Median	*p*	HR	95% CI	*p*
**Age**			0.279			
<60/≥60	84/87	230/193				
**Gender**			0.457			
Female/Male	37/134	353/208				
**Smoking history**			0.200			
Never smoker	49	213				
Ever smoker	18	154				
Current smoker + Unknown	104	225				
**DM/HT history**			0.577			
No/Yes	111/60	225/213				
**Histology**			0.757			
AC	133	208				
SCC	25	227				
Others^#^	13	250				
**Grade**			0.914			
Well + Moderately differentiated	7	202				
Poor + Undifferentiated differentiated	37	187				
Unknown	39	227				
**Mutation status**						**0.043**
*KRAS*	52	250		–	–	–
Driver gene	52	154		0.518	0.282–0.951	0.034
Wild-type	67	227		0.543	0.316–0.931	0.026
***TP53* co-mutation**			0.489			
No/Yes	125/46	227/187				
**PD-L1 expression level**				0.814	0.671–0.986	**0.036**
<1%	47	154				
1%-49%	31	215				
≥50%	31	NR				
Unknown	62	225				
**Brain metastases**			0.981			
No/Yes	143/28	215/176				
**Bone metastases**			0.866			
Single/Multiple	36/135	215/213				
**Visceral metastases**			**0.021**			
No/Yes	119/52	230/154				
**Treatment modality**			0.115			**0.042**
DI group	40	378		-	-	-
PI group	74	190		2.223	1.173–4.215	0.014
DnI group	15	170		2.785	1.113–6.971	0.029
PnI group	42	250		1.989	0.984–4.021	0.056
**Application line**			**0.006**	1.621	1.118–2.351	**0.011**
1	131	227				
2	29	125				
≥3	11	133				
**ECOG PS**			**0.001**	1.814	1.095–3.008	**0.021**
0	34	298				
1	124	225				
2	13	77				

DI, denosumab + ICIs; DnI, denosumab + non-ICIs; PI, phosphates + ICIs; PnI, phosphates + non-ICIs; DM, diabetes mellitus; HT, hypertension; AC, adenocarcinoma; SCC, squamous cell carcinoma; KRAS, Kirsten Rat Sarcoma Viral Oncogene Homolog; PD-L1, programmed death-ligand 1; ECOG PS, Eastern Cooperative Oncology Group performance status. *Current smoker refers to someone who has smoked more than 100 cigarettes (including hand-rolled cigarettes, cigars, cigarillos, etc.) in their lifetime and has smoked in the last 28 days. Ever smoker refers to someone who has smoked more than 100 cigarettes in their lifetime but has not smoked in the last 28 days. Never smoker is someone who has not smoked more than 100 cigarettes in their lifetime and does not currently smoke. *The log-rank test was employed for the comparative analysis. #The Cox’s proportional hazards regression model was used to analyze the influencing factors of PFS. Set the first subgroup for each variable as reference. ^#^Including large cell neuroendocrine carcinoma, sarcomatoid carcinoma, and adenosquamous carcinoma. Bold values indicate that the differences are statistically significant.

### 3.4 Toxicity evaluation

The most frequent AEs were pyrexia (12.3%) in the overall cohort, followed by fatigue (4.1%), arthralgia (3.5%), myalgia (3.5%), and renal failure (3.5%) **(** **Table 4** **)**. The PI group showed a trend for higher overall AEs than those of the DI, DnI, and PnI groups (39.2%, 27.5%, 26.7%, and 28.6%, respectively), though a significant difference was not reached (*p* = 0.742). The DI group demonstrated relatively comparable levels of AEs to the PnI group but fewer AEs than the PI group regarding pyrexia, arthralgia, myalgia, and renal failure.

**Table 4:** Treatment-emergent AEs between four treatment subgroups (n = 171).

AEs	Treatment modality/n (%)				Total/n (%)*
	DI group (n = 40)	PI group (n = 74)	DnI group (n = 15)	PnI group (n = 42)
Pyrexia	4 (10)	11 (14.9)	1 (6.7)	5 (11.9)	21 (12.3)
Bone pain	1 (2.5)	0	0	1 (2.3)	2 (1.2)
Arthralgia	1 (2.5)	4 (5.4)	0	1 (2.3)	6 (3.5)
Chills	0	0	0	0	0
Myalgia	1 (2.5)	4 (5.4)	0	1 (2.3)	6 (3.5)
Renal failure	1 (2.5)	3 (4.1)	1 (6.7)	1 (2.3)	6 (3.5)
Hypocalcemia	1 (2.5)	1 (1.4)	1 (6.7)	0	3 (1.8)
Toothache	0	2 (2.7)	0	1 (2.3)	3 (1.8)
Fatigue	1 (2.5)	3 (4.1)	1 (6.7)	2 (9.5)	7 (4.1)
Osteonecrosis of jaw	0	1 (1.4)	0	0	1 (0.6)
Feet numbness	1 (2.5)	0	0	0	1 (0.6)
**Total/n (%)**	11 (27.5)	29 (39.2)	4 (26.7)	12 (28.6)	56 (32.7)

DI, denosumab + ICIs; DnI, denosumab + non-ICIs; PI, phosphates + ICIs; PnI, phosphates + non-ICIs; AEs, adverse events. There were no fatal AEs. *Calculated as the percentage of the overall cohort.

## 4. Discussion

Based on previous studies, we further explored and confirmed the synergistic effects and safety of ICIs and denosumab. Through setting three parallel comparative subgroups, we found that concomitant therapy in the DI group was associated with better PFS and with a good safety profile.

The RANK–RANKL pathway is best known for its essential role in the biological and pathological processes of bone. RANKL produced by osteoblasts and bone marrow mesenchymal cells can attract aggregation of RANK-expressing cancer cells and induce migration of cancer cells through specific signaling cascade activation (especially the MAPK pathway), thus leading to bone metastasis formation and bone destruction (2). In addition to its bone-derived role, evidence has shown that it plays an important role in promoting tumor growth in a variety of malignancies and is confirmed as a worse prognostic factor (2, 4–7). In a *KRAS* ^G12D^-driven lung cancer model, Rao et al. (20) found that RANK expression appeared in the early stages of highly plastic tumor development, suggesting that RANK was a driver of early tumor progression. Further studies revealed that the complex interaction of the RANK/RANKL pathway and mitochondrial respiratory metabolism ultimately directly stimulated the proliferation of *KRAS* ^G12D^ mutant stem-like lung cancer cells through activation of the p38 and NF-κB pathways (20). Targeting RANKL seems to be a promising anti-tumor approach, but unfortunately, the randomized open-label phase III SPLENDOUR trial, which was designed to evaluate whether the addition of denosumab to standard first-line platinum-based doublet chemotherapy improved OS in advanced NSCLC, failed to demonstrate a clinical benefit (21).

However, besides cancer cells, RANK and RANKL are also expressed extensively in the TME, with RANK mainly on immature dendritic cells, immunosuppressive m2-type macrophages, and myeloid-derived suppressor cells, whereas RANKL mainly on CD8+ T cells (including regulatory T cells) (4). As a cytokine expressed on T cells, RANKL stimulates the survival of RANK-expressing dendritic cells (DCs) and enhances the ability of DCs to trigger the proliferation of naïve T cells (22). In the TME, RANKL interacts with RANK to coordinate various immunosuppressive processes through a variety of mechanisms (4). The 3LL lung adenocarcinoma mouse model constructed by Liede et al. showed that RANKL inhibitor combined with PD-1 mAb had a better therapeutic effect than RANKL inhibitor and PD-1 mAb alone (14). The study of Ahern et al. found that the combination of RANKL inhibitor and PD-1 mAb in a mouse model could further increase the number of infiltrating CD4^+^ and CD8^+^ T cells that can produce both IFN-γ and TNF in the TME, thus verifying the antitumor synergy effect of PD-1 mAb and RANKL inhibitor (12). However, in the early days, the immunomodulatory role of the RANK–RANKL pathway did not receive much attention or application until the development and application of denosumab.

Currently, as the first fully human anti-RANKL mAb, denosumab has been approved by the FDA for the prevention of SRE in solid tumors, including melanoma and lung cancer. For melanoma, in a retrospective study, Afzal and Shirai evaluated the synergistic effect of immune checkpoint inhibitors combined with denosumab in patients with metastatic melanoma, and the results showed that the PFS and OS of the group receiving the combination therapy were 11.6 months and 57 months, respectively, compared with 4.15 months and 22.8 months in the ICIs monotherapy group (10). For NSCLC, a retrospective clinical study that included 166 patients with advanced NSCLC by Liede et al. showed that continuous use of denosumab with ICIs significantly increased ORR (*p* <0.0001) and prolonged OS (*p* <0.0001) (14). Although the study of Liede et al. is very enlightening, due to the lack of an external control group, only patients with longer and shorter combination therapy within the study cohort were compared. This may lead to a reversal in deriving causality and consequent unfirm conclusion, because patients who were able to receive a longer duration of combination therapy (with better ORR and longer OS) may themselves be sensitive to immunotherapy, rather than as a result of the combination of denosumab. The same concern is also present in the study by Cao et al. (16). Bongiovanni et al. circumvented this by setting both the control group (immunotherapy monotherapy) and the experimental group (denosumab/ZA + immunotherapy). However, treatment with ZA was confounded in the study arm, so the synergistic effect of denosumab and immunotherapy could not be accurately assessed. Furthermore, it is possible that the additional prolongation of survival in the experimental group shown by the study results was due to the survival benefit of BTT by reducing SREs rather than synergy with immunotherapy (15). A summary of published data on the combination of denosumab and ICIs is demonstrated in **Table 5**.

**Table 5:** Previously published data on combination of denosumab and ICIs.

Author	Region	Year	Study Design	Comparison arms	Study Scale (n)	Histology (n/%)	First Line (n/%)	PD-1/PD-L1	BTT (n/%)	≥50% PD-L1 Expression (n/%)	ORR* (%)	PFS	OS
Bongiovanni (15)	Italy	2021	Retrospective	ICIs vs. ICIs + D/ZA	46	44 (95.7) AC	10 (21.7)	Nivolumab, Pembrolizumab, Atezolizumab	6 (20.0) with D; 24 (80.0) with ZA	NA	7.6 vs. 64.6^#^	4.0 vs. 7.1 months	15.8 vs. 21.8 months
Cao (16)	USA	2021	Retrospective	Duration of ICIs and D overlap (<3 vs. >3 months)	69	69 (100) AC	32 (46.4)	Pembrolizumab, Atezolizumab, Ipilimumab+Nivolumab	69 with D	16 (23.2)	3.88 vs. 14.92, overall 18.8	1.9 vs.6.0 months	3.6 vs. 11.5 months
Liede (14)	USA	2018	Retrospective	Duration of ICIs and D overlap (0–6 vs. 6–14 vs. >14 weeks)	166	105 (63.3) Non-squamous	37 (22.3)	Nivolumab Pembrolizumab Ipilimumab	166 with D	NA	No comparison data available, overall 33.1	NA	8.3 vs. 16.6 vs. 19.9 weeks
Ferrara (17)^	Italy	2021	Retrospective	ICIs vs. ICIs + D	49	NA	NA	NA	16 with D	NA	NA	1.8 vs. 2.6 months	3.5 vs. 15.1 months
				ICIs vs. ICIs + ZA	62	NA	NA	NA	29 with ZA	NA	NA	NA	3.8 vs. 3.5 months
Tsuchiya (18)	Japan	2022	Retrospective	ICIs vs. ICIs + D/ZA	29	22 (75.9) AC	8 (27.6)	Nivolumab, Pembrolizumab, Atezolizumab	20 (95.2) with D; 1 (3.4) with ZA	9 (36.0)	0 vs. 15; 0 vs. 35#	NA	2.5 vs. 16.0 months
Present study	China	2022	Retrospective	DI vs. PI vs. DnI vs. PnI	171	133 (77.8) AC	131 (76.6)	Pembrolizumab, Nivolumab, Atezolizumab, Sintilimab, Camrelizumab	55 (32.2) with D; 116 (67.8) with P	31 (18.1)	47.5 vs. 43.2 vs. 33.3 vs. 40.5	378 vs. 190 vs. 170 vs. 172 days	NA

ICIs, immune checkpoint inhibitors; AC, adenocarcinoma; BTT, bone targeted agents; PD-1, programmed death 1; PD-L1, programmed death ligand 1; D, denosumab; P, phosphates; ZA, zoledronic acid; DI, denosumab + ICIs; DnI, denosumab + non-ICIs; PI, phosphates + ICIs; PnI, phosphates + non-ICIs; ORR, objective response rate; PFS, progression-free survival; OS, overall survival; NA, not available. * Per RECIST 1.1. ^#^ Per MDA criteria. ^ Analyses were performed on patients with high bone tumor burden ≥3 bone metastases at ICIs baseline.N/A, Not available.

Therefore, to exclude the effect of confounding factors and to enhance the persuasiveness of the study, we set up one experimental group and three control groups. Besides, we also explored the effect of mutation status on the efficacy of combination therapy. The results of the study suggested an improved PFS for the DI group compared with the PI group in the overall cohort (*p* = 0.115), which was more pronounced in the WILD-TYPE cohort (*p* = 0.125) and the NON-DRIVER cohort (*p* = 0.045). Meanwhile, we did not observe significant differences in PFS between the DnI and PnI groups. Hence, it is feasible to assume that the improved efficacy of denosumab over ZA originated from a synergistic effect with ICIs rather than from a difference in the ability to reduce SREs between denosumab and ZA. Our study results were partly supported by previous data from Bongiovanni et al. (15), whose work indicated a better mPFS (15.9 months; 95%CI, 5.1–not estimable) of patients receiving denosumab (n = 6) than those treated with ICIs alone or with ZA (*p* = 0.068).

Two prospective studies concerning the DI treatment of lung cancer are currently underway. The DENIVOS (NCT03669523) study aims to evaluate the combination of denosumab and nivolumab in the second line of NSCLC with bone metastases. The POPCORN study (ACTRN12618001121257) is designed to provide information about the activity and safety of the combination of denosumab and nivolumab compared with nivolumab alone in the preoperative treatment of resectable NSCLC (23). Hopefully, these studies will shed light on the future exploration and application of the combination of denosumab and ICIs in NSCLC.

This study has some inherent limitations. First, as this study was a single-center study, and the included patients were mainly from urban areas, there was a selection bias. Second, the number of patients in individual groups in the study cohort was too small, which may affect the statistical test validity. Third, there were confounding factors in the study, such as different brands of PD-1/PD-L1 inhibitors and phosphates. In addition, patients receiving denosumab may not have been randomly selected because some physicians may prefer denosumab to phosphates, and the relatively higher cost of denosumab may also be an important factor in deciding whether a patient will receive denosumab or not. Although these influencing factors were not assessed in this study, they may still have influenced the outcomes. Finally, potential efficacy biomarkers, including RANKL and RANK, were not investigated. All of these have the potential to affect the reliability of the study results. Therefore, the results of the study should be interpreted with caution.

In conclusion, our findings suggest a synergistic effect of denosumab in combination with ICIs in the treatment of NSCLC patients carrying bone metastases, and this combination has a good safety profile.

## Data availability statement

The original data presented in the study are included in the article/supplementary material. Further inquiries can be directed to the corresponding authors.

## Ethics statement

The studies involving human participants were reviewed and approved by the Ethics Committee of National Cancer Center/Cancer Hospital, Chinese Academy of Medical Sciences and Peking Union Medical College. The patients/participants provided their written informed consent to participate in this study.

## Author contributions

Conception and design: H-SL, S-YL, H-YX, and YW. Administrative support: H-SL, JLL, PYX, XZH, HYX, and YW Provision of study materials or patients: J-LL, P-YX, X-ZH, HYX, and YW. Collection and assembly of data: H-SL and S-YL. Data analysis and interpretation: H-SL, S-YL, JLL, PYX, XZH, FX, and HYX Manuscript writing: All authors. Final approval of manuscript: All authors. All authors contributed to the article and approved the submitted version.

## Funding

This work was supported by the National Natural Science Foundation of China (Grant No. 82072590) and the Beijing Health Promotion Association (Grant No. 2021-053-ZZ).

## Acknowledgments

We would like to thank the support of all enrolled patients and their relatives, and we would also like to thank Editage (www.editage.cn) for English language editing.

## Conflict of interest

The authors declare that the research was conducted in the absence of any commercial or financial relationships that could be construed as a potential conflict of interest.

## Publisher’s note

All claims expressed in this article are solely those of the authors and do not necessarily represent those of their affiliated organizations, or those of the publisher, the editors and the reviewers. Any product that may be evaluated in this article, or claim that may be made by its manufacturer, is not guaranteed or endorsed by the publisher.
